# B cells regulate thymic CD8^+^T cell differentiation in lupus-prone mice

**DOI:** 10.18632/oncotarget.19002

**Published:** 2017-07-05

**Authors:** Chen Xing, Gaizhi Zhu, He Xiao, Ying Fang, Xiaoling Liu, Gencheng Han, Guojiang Chen, Chunmei Hou, Beifen Shen, Yan Li, Ning Ma, Renxi Wang

**Affiliations:** ^1^ Laboratory of Immunology, Institute of Basic Medical Sciences, Beijing, China; ^2^ Laboratory of Cellular and Molecular Immunology, Henan University, Kaifeng, Henan, China; ^3^ Department of Rheumatology, First hospital of Jilin University, Changchun, China; ^4^ Department of Nephrology, The 307th Hospital of Chinese People’s Liberation Army, Beijing, China; ^5^ Department of Stress Medicine, Beijing Institute of Basic Medical Sciences, Beijing, China

**Keywords:** B cells, thymic CD8^+^T cells, RORγt, IgG, lupus-prone mice, Immunology and Microbiology Section, Immune response, Immunity

## Abstract

Previous studies have shown that under normal physiological conditions thymic B cells play a critical function in T cell negative selection. We tested the effect of thymic B cells on thymic T-cell differentiation in autoimmune diseases including systemic lupus erythematosus (SLE). We found that thymic B cells and CD8^-^ CD4^+^ and CD4^-^CD8^+^T cells increased, whereas CD4^+^CD8^+^T cells decreased in lupus-prone mice. Once B cells were reduced, the change was reversed. Furthermore, we found that B cells blocked thymic immature single positive (ISP) CD4^-^CD8^+^CD3^lo/-^RORγt^-^ T cells progression into CD4^+^CD8^+^T cells. Interestingly, we found a novel population of thymic immature T cells (CD4^-^CD8^+^CD3^lo^RORγt^+^) that were induced into mature CD4^-^CD8^+^CD3^+^RORγt^+^T cells by B cells in lupus-prone mice. Importantly, we found that IgG, produced by thymic B cells, played a critical role in the differentiation of thymic CD8^+^ISP and mature RORγt^+^CD8^+^ T cells in lupus-prone mice. In conclusion, B cells blocked the differentiation from thymic CD8^+^ISP and induced the differentiation of a novel immature CD4^-^CD8^+^CD3^lo^RORγt^+^T cells into mature RORγt^+^CD8^+^ T cells by secreting IgG antibody in lupus-prone mice.

## INTRODUCTION

Systemic lupus erythematosus (SLE) is a prototypical autoimmune disease characterized by B cell hyper-reactivity, abundant production of autoantibodies, and subsequent formation of immune complexes leading to tissue damage [1, 2]. MRL/lpr mice display autoimmunity and lymphoproliferation disease and are considered as a model of human SLE diseases [3, 4]. Although the pathogenesis of SLE remains unclear, the prevalence of autoantibodies early on before clinical symptoms of SLE are found implicates B cell dysregulation as a contributing factor to disease [5, 6]. In addition, B-cell-targeting drugs such as Belimumab and Rituximab, have proven to be extremely effective in SLE patients [7–9] suggesting that B cell dysregulation plays a critical role in the pathogenesis of SLE.

Apart from B cells, autoreactive T cells play significant roles in SLE disease pathogenesis [10]. Recent compelling evidence has suggested that T cells are crucial in the pathogenesis of SLE, enhancing the production of autoantibodies by offering substantial help to B cells by stimulating their differentiation, proliferation, and maturation, in addition to their support for class-switching of autoantibodies [11]. In addition, the immunological characteristics of the infiltrating CD4^+^ and CD8^+^ T cells in the lupus kidney indicate they have the potential to mediate injury [12]. Urinary T-cells, in particular CD8^+^ T cells, are a promising marker to assess renal activity in patients with lupus nephritis, in particular in those with prior renal involvement [13]. Furthermore, the CD4^+^/CD8^+^ T cell ratio decreased in SLE patients [14] and there was a marked increase in the frequency and functional activity of Th17, Tc17 and other T-cell subsets in active compared to inactive SLE [15].

The thymus is the indispensable organ for T cell development in mammals. After the CD4^-^CD8^-^ double-negative (DN) stages, thymocytes express CD8 and become immature single-positive (ISP) CD8^+^ (for mice) or CD4^+^ (for human) thymocytes before expressing CD4 and becoming CD4^+^CD8^+^ double positive (DP) thymocytes [16, 17]. These DP cells are subjected to positive and negative selection culminating in a relatively small number of CD4^+^CD8^-^ and CD4^-^CD8^+^ ‘single positive’ (SP) thymocytes [18, 19]. The thymus has a main function in establishing and sustaining immunological self-tolerance; i.e., negative selection (clonal deletion) of self-reactive T cells [20]. Thymic generation of autoimmune T cells leads to activated, expanded, and differentiated autoimmune T cells in the periphery. A common characteristic of many autoimmune diseases models is the generation of a peripheral T-cell repertoire containing a large number of autoreactive T cells because of the impaired thymic negative selection [21].

A small population of B cells make up around 0.1-0.5% of thymocytes in both humans and mice [22, 23]. Thymic B cells preferentially reside at the junction of the thymic cortex and the medulla, an area where negative selection is thought to occur. We and other researchers have shown that thymic B cells play a critical function in T cell negative selection [22–24]. Thus, we determined whether thymic B cells increased and regulated thymic T cell differentiation in autoimmune diseases like SLE.

## RESULTS

### Lupus up-regulated thymic B cells, CD4^+^CD8^-^ and CD4^-^CD8^+^T cells, and reduced CD4^+^CD8^+^T cells

**Figure 1:**
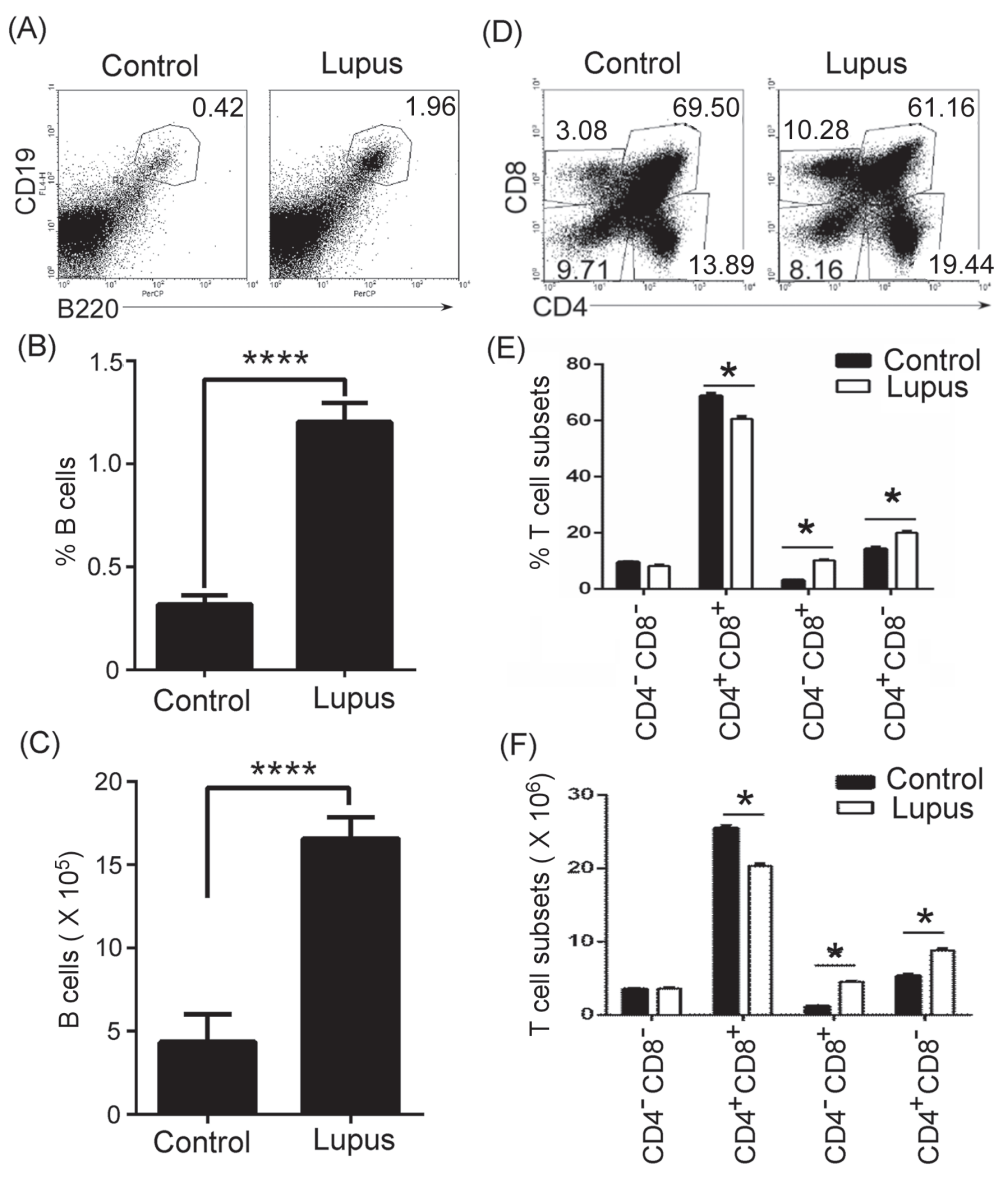
Thymic B cells and CD4 ^+^CD8^-^ and CD4^-^CD8^+^T cells increased in lupus-prone mice **A.**, **B.**, **C.** Thymic B cells increased in lupus-prone mice. Single-cell suspension of thymocytes from 7-9-week-old non-lupus-prone MRL/+ (Control) and lupus-prone MRL/lpr (Lupus) mice (6 mice per group) was obtained simply by mechanical disruption. Thymocytes were stained with anti-mouse B220 and CD19 antibody, and analyzed by flow cytometry (FACS). The percentage **A.**, the statistical results for the percentage **B.**, and the absolute numbers **C.**, of thymic B cells are shown. **D.**, **E.**, **F.** Thymic CD4^+^CD8^-^ and CD4^-^CD8^+^T cells increased in lupus-prone mice. Thymocytes as described in Figure 1A-C were stained with anti-mouse CD4 and CD8 antibodies and analyzed by FACS. The percentage **D.**, the statistical results for the percentage **E.**, and the absolute numbers **F.**, of thymic CD4^-^CD8^-^ and CD4^+^CD8^+^T, CD4^+^CD8^-^ and CD4^-^CD8^+^T cells are shown. **B.**, **C.**, **E.**, **F.** Data are shown as mean + SEM (*n* = 18) from three independent experiments. *P < 0.05, ****P < 0.0001. **B.**, **C.** Two tailed student's *t*-test; **E.**, **F.** Two-Way ANOVA plus Bonferroni post-tests compared each column *vs* control column. Error bars, s.e.m.

To determine whether lupus up-regulated thymic B-cell numbers, thymocytes were isolated from 7-9-week-old non-lupus-prone MRL/+ and lupus-prone MRL/lpr mice. Flow cytometry (FACS) analysis demonstrated that the percentages and the absolute numbers of thymic B cells increased in lupus-prone mice (Figure 1A-1C). In accordance with the changes in thymic B cells, the percentages and the absolute numbers of thymic CD4^+^CD8^-^ and CD4^-^CD8^+^T cell also increased in lupus-prone mice (Figure 1D-1F). Unexpectedly, we found that CD4^+^CD8^+^T cells were reduced in lupus-prone mice (Figure 1D-1F). Together, these data suggest that lupus regulated the change of thymic B and T cells.

**Table I:** Highly expressed surface marker in thymic CD4^-^CD8^+^CD3^lo/-^T cells

Gene	Description	Fold change(CD3^hi^/CD3^lo/-^)	
Tnfrsf8	tumor necrosis factor receptor superfamily, member 8	0.3	down
Il12rb2	interleukin 12 receptor, beta 2	0.33	down
Fcgr2b	Fc receptor, IgG, low affinity IIb	0.35	down
Fcer2a	Fc receptor, IgE, low affinity II, alpha polypeptide	0.37	down
Ptcra	pre T cell antigen receptor alpha	0.39	down
Socs4	suppressor of cytokine signaling 4	0.45	down
Cxcr5	chemokine (C-X-C motif) receptor 5	0.45	down

The transcripts in thymic CD4^-^CD8^+^CD3^lo/-^ and CD4^-^CD8^+^CD3^+^T cells were determined by Affymetrix microarrays. Most significantly changed mRNA transcripts are shown.

### Thymic B cells positively regulated thymic CD4^-^CD8^+^T cells

**Figure 2:**
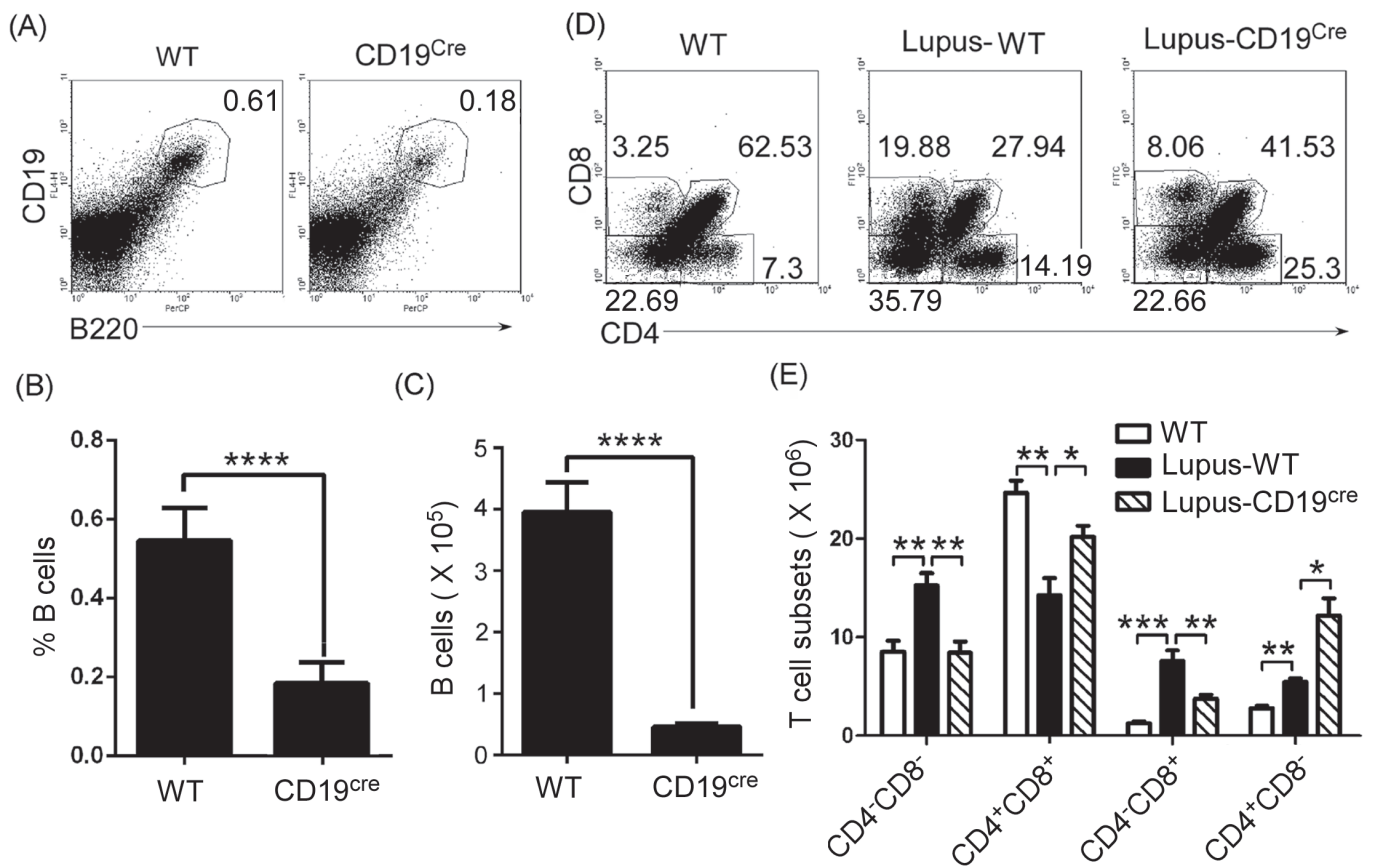
Thymic CD4 ^-^CD8^+^T cell numbers decreased in B cells-reduced mice **A.**, **B.**, **C.** Thymic B cells decreased in homozygous CD19^cre^ (CD19-deficient) mice. A single-cell suspension of thymocytes from 7-9-week-old wild type (WT) C57BL/6 mice and homozygous CD19^cre^ mice on the background of C57BL/6 mice (6 mice per group) was obtained simply by mechanical disruption. Thymocytes were stained with anti-mouse B220 and CD19 antibody and analyzed by FACS. The percentage **A.**, the statistical results for the percentage **B.**, and the absolute numbers **C.**, of thymic B cells are shown. **D.**, **E.** Thymic CD4^-^CD8^+^T cells decreased in B cells-reduced mice. 0.5 ml the lupus-inducing compound pristane (2,6,10,14-Tetramethylpentadecane or TMPD) per mouse was injected i.p. into WT and homozygous CD19^cre^ mice (6 mice per group). On day 21 after injection, thymocytes were collected as described in Figure 2A-2C, stained with anti-mouse CD4 and CD8 antibodies, and analyzed by FACS. The percentage **D.**, and the absolute numbers **E.**, of thymic CD4^-^CD8^-^ and CD4^+^CD8^+^T, CD4^+^CD8^-^ and CD4^-^CD8^+^T cells are shown. **B.**, **C.**, **E.** Data are shown as mean + SEM (n = 18) from three independent experiments. *P < 0.05, **P < 0.01, ***P < 0.001, ****P < 0.0001. **B.**, **C.** Two tailed student's *t*-test; **E.** Two-Way ANOVA plus Bonferroni post-tests were used to compare each column *vs* control (Lupus-WT) column. Error bars, s.e.m.

To detect the effect of thymic B cells on thymic T-cell differentiation, we needed B cell-deficient or -reduced mice. First, we determined the level of thymic B cells in homozygous CD19^cre^ (CD19-deficient) mice. Thymocytes were isolated from 7-9-week-old wild type (WT) and CD19-deficient mice. FACS analysis demonstrated that the percentages and the absolute numbers of thymic B cells were significantly reduced in CD19-deficient mice (Figure 2A-2C). These data suggest that homozygous CD19^cre^ mice substitute for thymic B-cell-reduced mice. To assess the effect of thymic B cells on thymic T-cell differentiation, we analyzed thymic CD4^-^CD8^-^, CD4^+^CD8^+^, CD4^+^CD8^-^ and CD4^-^CD8^+^ T cell percentage and absolute numbers. We found that thymic CD4^+^CD8^+^ T cells increased, whereas CD4^-^CD8^-^ and CD4^-^CD8^+^ T cells reduced in homozygous CD19^cre^ mice (Supplementary Figure S1A and S1B). Importantly, in homozygous CD19^cre^ mice, thymic B cells mainly regulated thymic CD4^-^CD8^+^ but not CD4^+^CD8^-^ T cells in lupus-induced mice (Supplementary Figure S1A and S1B).

To assess the effect of thymic B cells on thymic T-cell differentiation in autoimmune diseases, we injected lupus-inducing pristane [25] into homozygous CD19^cre^ (CD19-deficient) mice. In accordance with the data in lupus-prone mice, lupus-inducing pristane up-regulated the thymic CD4^+^CD8^-^ and CD4^-^CD8^+^T cell percentage and absolute numbers and reduced CD4^+^CD8^+^T cells (Figure 2D and 2E). Critically, we found that in homozygous CD19^cre^ mice, lupus-inducing pristane did not up-regulate thymic CD4^-^CD8^+^ but up-regulated CD4^+^CD8^-^ T cells (Figure 2D and 2E). The data suggest that thymic B cells mainly regulated thymic CD4^-^CD8^+^ but not CD4^+^CD8^-^ T cells in lupus-induced mice.

Our previous studies have shown that atacicept (TACI-IgG) effectively reduces B cells in lupus-prone mice by binding a portion of the receptor TACI to block the effects of survival factors BAFF (B-cell activation factor) and a proliferating-inducing ligand (APRIL) [26]. We found here that TACI-IgG could also effectively reduce thymic B cells in lupus-prone MRL/lpr mice (Supplementary Figure S2A-S2C). Accordingly, thymic B-cell reduction reduced thymic CD4^-^CD8^+^ but not CD4^+^CD8^-^ T cell numbers in lupus-prone MRL/lpr mice (Supplementary Figure S2D and S2E).

Altogether, these results suggest that thymic B-cell reduction may initiate the thymic CD4 or CD8 lineage ‘decision’ in lupus-prone and pristane-treated mice.

### Peripheral mature CD8^+^ and RORγt^+^CD8^+^ T cells increased in lupus-prone mice

**Figure 3:**
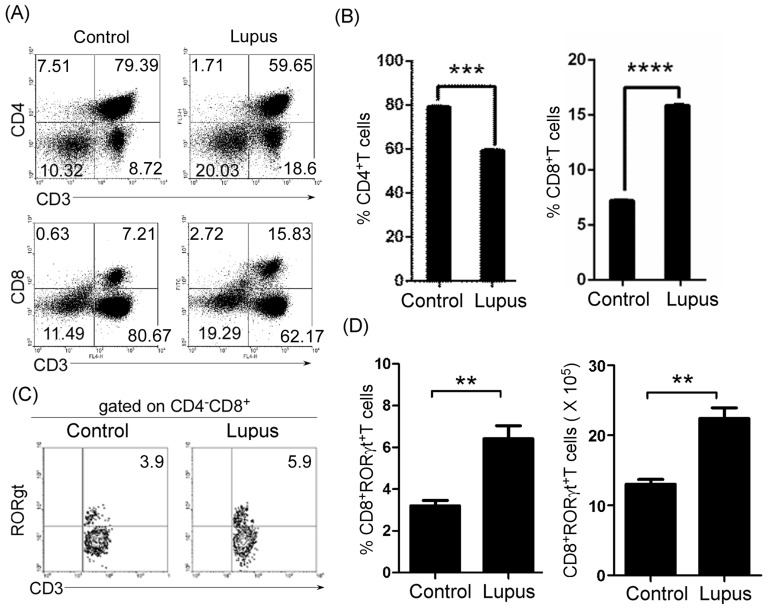
Peripheral mature CD8 ^+^ and RORγt^+^CD8^+^ T cells increased in lupus-prone mice **A.**, **B.** The ratio of peripheral CD8^+^ to CD4^+^ T cells increased in lupus-prone mice. Lymphocytes were separated from lymph nodes of 7-9-week-old non-lupus-prone MRL/+ (Control) and lupus-prone MRL/lpr (Lupus) mice (6 mice per group). Lymphocytes were stained with anti-mouse CD3, CD4, and CD8 antibodies, and analyzed by FACS. **A.** Quadrants indicate percentage of CD3^+^, CD4^+^ and CD8^+^ T cells. **B.** The statistical results for the percentage of CD4^+^ and CD8^+^ T cells are shown. **C.**, **D.** Peripheral RORγt^+^CD8^+^ T cells increased in lupus-prone mice. Lymphocytes from lymph nodes of 7-9-week-old non-lupus-prone MRL/+ (Control) and lupus-prone MRL/lpr (Lupus) mice (6 mice per group) were stained with anti-mouse CD3, CD8, and RORγt antibodies, and analyzed by FACS. **C.** Quadrants indicate percentage of RORγt-expressing CD3^+^T of CD4^-^CD8^+^ T cells. **D.** The statistical results for the percentage (Left panel) and the absolute numbers (Right panel) of CD3^+^CD4^-^RORγt^+^CD8^+^ T cells are shown. **B.**, **D.** Data are shown as mean + SEM (*n* = 24) from four independent experiments. *P < 0.05, **P < 0.01, ***P < 0.001, ****P < 0.0001. Two tailed student's *t*-test. Error bars, s.e.m.

Next, we determined the level of peripheral mature CD8^+^ and RORγt^+^CD8^+^ T cells in lupus-prone mice. Lymphocytes from the lymph nodes of 7-9-week-old non-lupus-prone MRL/+ and lupus-prone MRL/lpr mice were stained with anti-mouse CD3, CD4, CD8, and RORγt antibodies, and analyzed by FACS. We found that the ratio of peripheral CD8^+^ to CD4^+^ T cells increased in lupus-prone (Figure 3A and 3B) and pristane-treated (Supplementary Figure S3A and S3B) mice. In addition, we also found that peripheral RORγt^+^CD8^+^ T cells increased in lupus-prone (Figure 3C and 3D) and pristane-treated (Supplementary Figure S3C and S3D) mice. Importantly, B-cell reduction could effectively reverse the increased ratio of peripheral CD8^+^ to CD4^+^ T cells and the increase in RORγt^+^CD8^+^ T cells (Supplementary Figure S3A-S3D). The T cell specific isoform of RORγ, known as RORγt, drives the activation and differentiation of CD4^+^ and CD8^+^ cells into IL17-producing helper T cells (Th17) and cytotoxic T cells (Tc17) [27, 28]. In accordance with increased RORγt^+^CD8^+^ T cells, IL-17-producing Tc17 increased in pristane-treated mice (Supplementary Figure S3E and S3F). Critically, B-cell reduction could effectively reverse an increase in IL-17-producing Tc17 cells (Supplementary Figure S3E and S3F). These results suggest that thymic B cells may control the ratio of peripheral CD8^+^ to CD4^+^ T cells and Tc17 cells by regulating the thymic CD4 or CD8 lineage ‘decision’.

### Thymic B cells controlled immature single positive (ISP) CD8^+^T cell differentiation in lupus-prone mice

**Figure 4:**
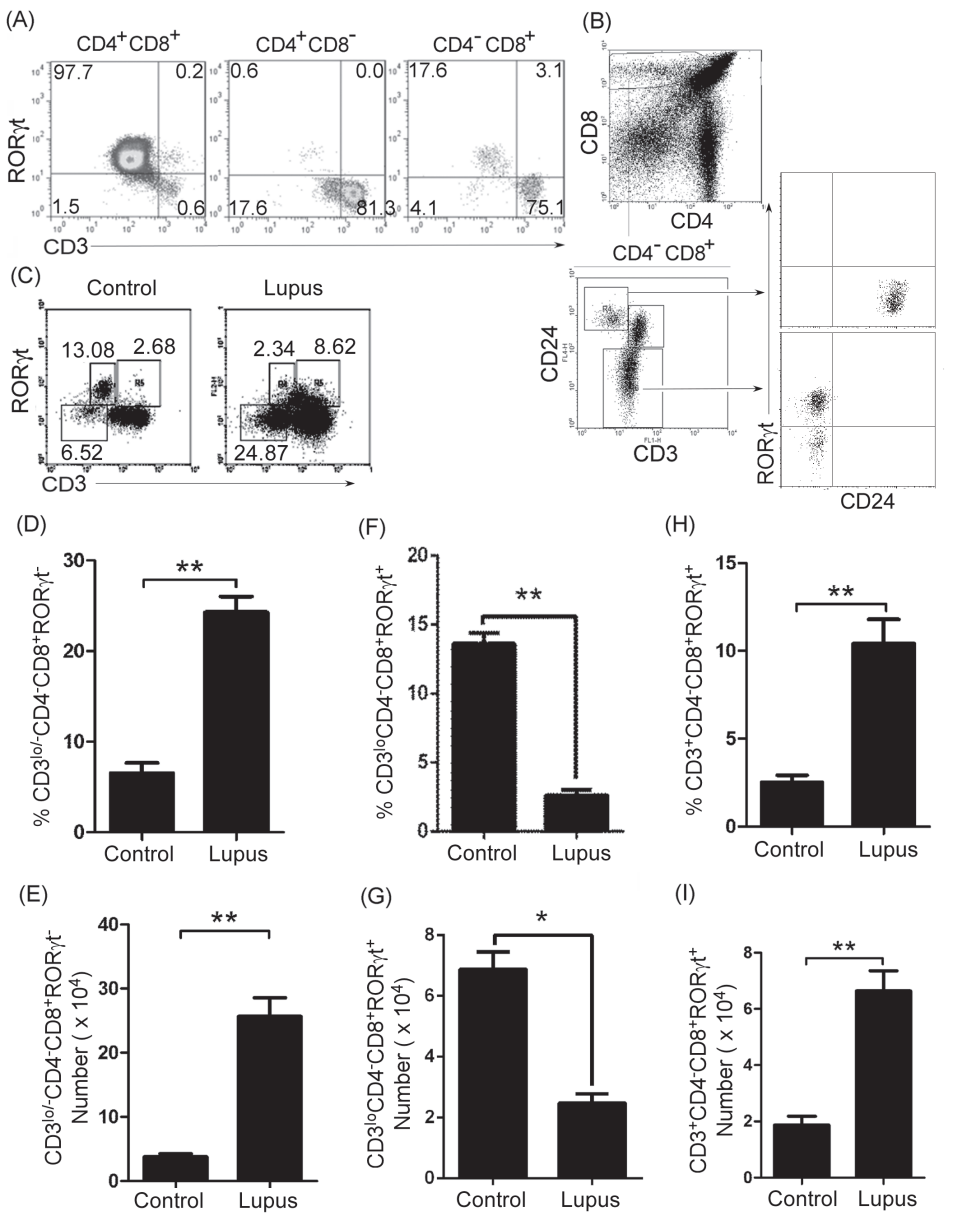
Thymic immature single positive (ISP) CD4 ^-^CD8^+^CD3^lo/-^RORγt^-^ T cells and mature CD4^-^CD8^+^CD3^+^RORγt^+^T cells increased in lupus-prone mice **A.**, **B.** Thymic ISP CD8^+^T cells expressed high levels of CD24 but not RORγt. A single-cell suspension of thymocytes from 7-9-week-old C57BL/6 mice (6 mice) was obtained simply by mechanical disruption. Thymocytes were stained with anti-mouse CD4, CD8, CD3, CD24 and RORγt antibody and analyzed by flow cytometry (FACS). **A.** The percentage of RORγt- and/or CD3-expressing cells on gated CD4^+^CD8^+^, CD4^+^CD8^-^ and CD4^-^CD8^+^ T cells, **B.** RORγt and CD24 expression (lower and right panel) in CD3^lo^CD24^hi^ and CD3^mi^CD24^-^ cells (lower and left panel) on gated CD4^-^CD8^+^ T cells (upper panel) are shown. **C.**-**I.** Thymic ISP CD8^+^T cells increased, immature CD4^-^CD8^+^CD3^lo^RORγt^+^T cells decreased, and mature CD4^-^CD8^+^CD3^+^RORγt^+^T cells increased in lupus-prone mice. A single-cell suspension of thymocytes from 7-9-week-old non-lupus-prone MRL/+ (Control) and lupus-prone MRL/lpr (Lupus) mice (6 mice per group) was obtained simply by mechanical disruption. Thymocytes were stained with anti-mouse CD3, CD4, CD8 and RORγt antibodies, and analyzed by FACS. The percentage **C.**, the statistical results for the percentage **D.**, **F.**, **H.**, and the absolute numbers **E.**, **G.**, **I.**, of thymic CD4^-^CD8^+^CD3^lo/-^RORγt^-^ ISP D., E., CD4^-^CD8^+^CD3^lo^RORγt^+^ **F.**, **G.**, and CD4^-^CD8^+^CD3^+^RORγt^+^ **H.**, **I.**T cells are shown. **D.**-**I.** Data are shown as mean + SEM (*n* = 18) from three independent experiments. *P < 0.05, **P < 0.01. Two tailed student's *t*-test. Error bars, s.e.m.

To further determine which population of thymic CD4^-^CD8^+^ T cells was regulated by thymic B cells in lupus-prone mice, we first identified CD4^-^CD8^+^ T cell phenotype by analyzing CD3, RORγt, and CD24 expression in C57BL/6 (Figure 4A and 4B) and MRL/+ (Supplementary Figure S4A and S4B) mice. We found three different populations (CD4^-^CD8^+^CD3^lo/-^RORγt^-^T, CD4^-^CD8^+^CD3^lo^RORγt^+^T, and CD4^-^CD8^+^CD3^+^RORγt^+^T) of CD4^-^CD8^+^ T cells (Figure 4A and Supplementary Figure S4A). Previous studies have shown that immature single-positive (ISP) CD8^+^ thymocytes are CD4^-^CD8^+^CD3^lo/-^CD24^hi^T cells [16, 17]. Next, we determined the level of CD24 in CD4^-^CD8^+^CD3^lo/-^RORγt^-^T and CD4^-^CD8^+^CD3^lo^RORγt^+^T cells. We found that CD4^-^CD8^+^CD3^lo/-^RORγt^-^T cells expressed a high level of CD24, whereas CD4^-^CD8^+^CD3^lo^RORγt^+^T cells expressed a moderate level of CD24 (Figure 4B and Supplementary Figure S4B). These results suggest that CD4^-^CD8^+^CD3^lo/-^RORγt^-^T cells were ISP CD8^+^ thymocytes.

We found that thymic ISP CD8^+^T cells increased in lupus-prone mice (Figure 4C-4E). In addition, we also found that thymic B cells up-regulated CD4^-^CD8^-^ T cells and reduced CD4^+^CD8^+^T cells (Figure 2D and 2E). Thus, we propose that thymic B cells might block thymic ISP CD8^+^T cell differentiation so that CD4^-^CD8^-^ T cells were up-regulated and CD4^+^CD8^+^T cells were reduced.

To assess the effect of thymic B cells on ISP CD8^+^T cells, we examined CD4^-^CD8^+^CD3^lo/-^RORγt^-^ ISP CD8^+^T cells in homozygous CD19^cre^ mice. The data demonstrated that thymic ISP CD8^+^T cells decreased in homozygous CD19^cre^ mice, (Supplementary Figure S1C and S1D). These results suggest that thymic B cells may block thymic ISP CD8^+^T-cell differentiation.

**Figure 5:**
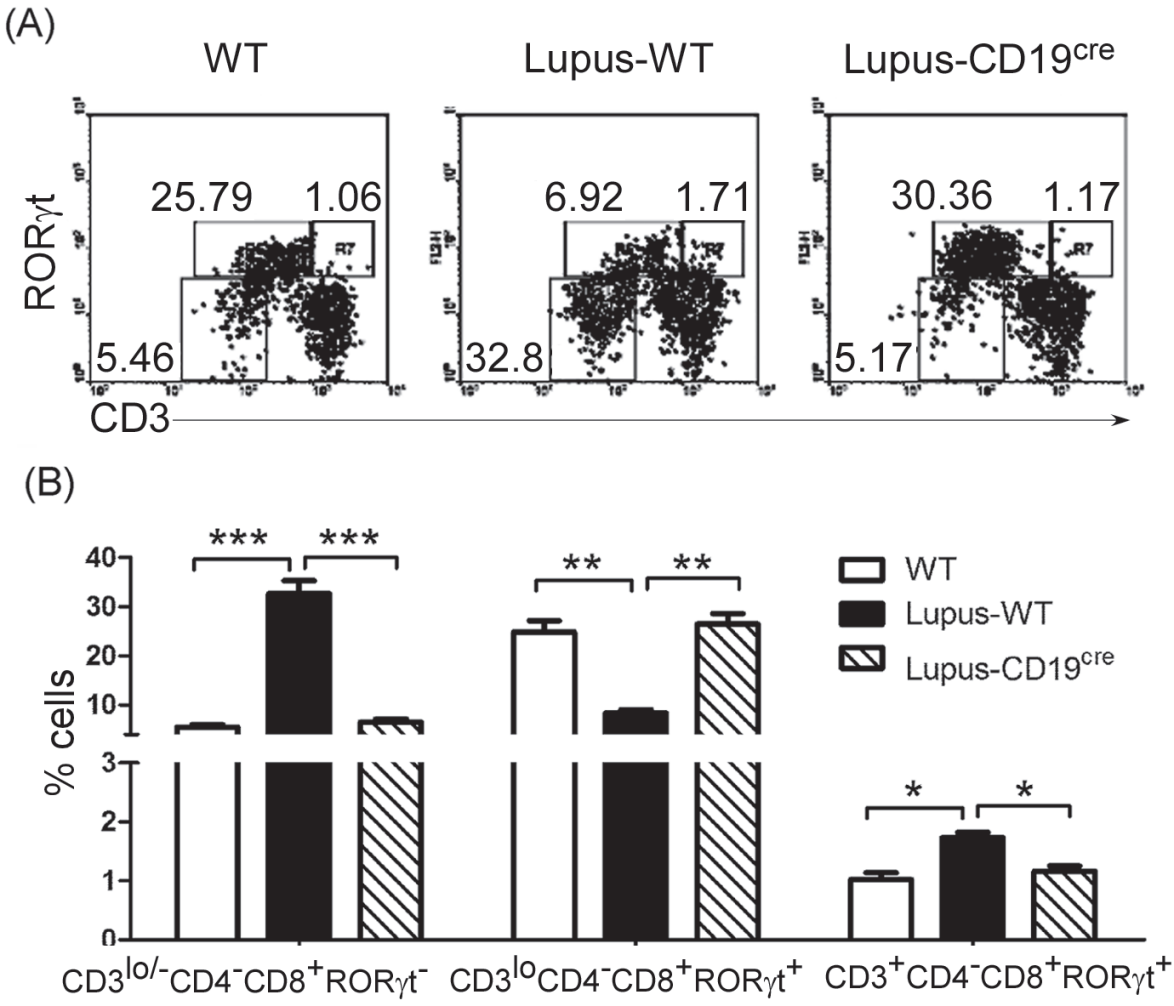
B-cell reduction reduced thymic CD4 ^-^CD8^+^CD3lo/-RORγt^-^ ISP T cells and mature CD4^-^CD8^+^CD3^+^RORγt^+^T cells and up-regulated immature CD4^-^CD8^+^CD3loRORγt^+^T cells 0.5 ml the lupus-inducing compound pristane per mice was injected i.p. into WT and homozygous CD19^cre^ mice (6 mice per group). On day 21 after injection, thymocytes were collected as described in Figure 2A-C, stained with anti-mouse CD4, CD8, CD3 and RORγt antibodies, and analyzed by FACS. The percentage **A.**, and the statistical results for the percentage **B.**, of thymic CD4^-^CD8^+^CD3^lo/-^RORγt^-^ ISP, CD4^-^CD8^+^CD3^lo^RORγt^+^ and CD4^-^CD8^+^CD3^+^RORγt^+^ T cells are shown. **B.** Data are shown as mean + SEM (*n* = 18) from three independent experiments. *P < 0.05, **P < 0.01, ***P < 0.001. Two-Way ANOVA plus Bonferroni post-tests compared each column *vs* control (Lupus-WT) column. Error bars, s.e.m.

To assess the effect of thymic B cells on ISP CD8^+^T cells in autoimmune diseases, we injected lupus-inducing pristane into homozygous CD19^cre^ mice. Lupus-inducing pristane up-regulated thymic ISP CD8^+^T cells (Figure 5A and 5B). Critically, we found that in homozygous CD19^cre^ mice, lupus-inducing pristane did not up-regulate thymic ISP CD8^+^T cells (Figure 5A and 5B). In accordance with these results, we found that B-cell reduction with TACI-IgG reduced thymic CD4^-^CD8^+^CD3^lo/-^RORγt^-^ ISP T cells in lupus-prone MRL/lpr mice (Supplementary Figure S2F and S2G). These data suggest that thymic B cells block thymic ISP CD8^+^T-cell differentiation in lupus-induced or -prone mice.

**Figure 6:**
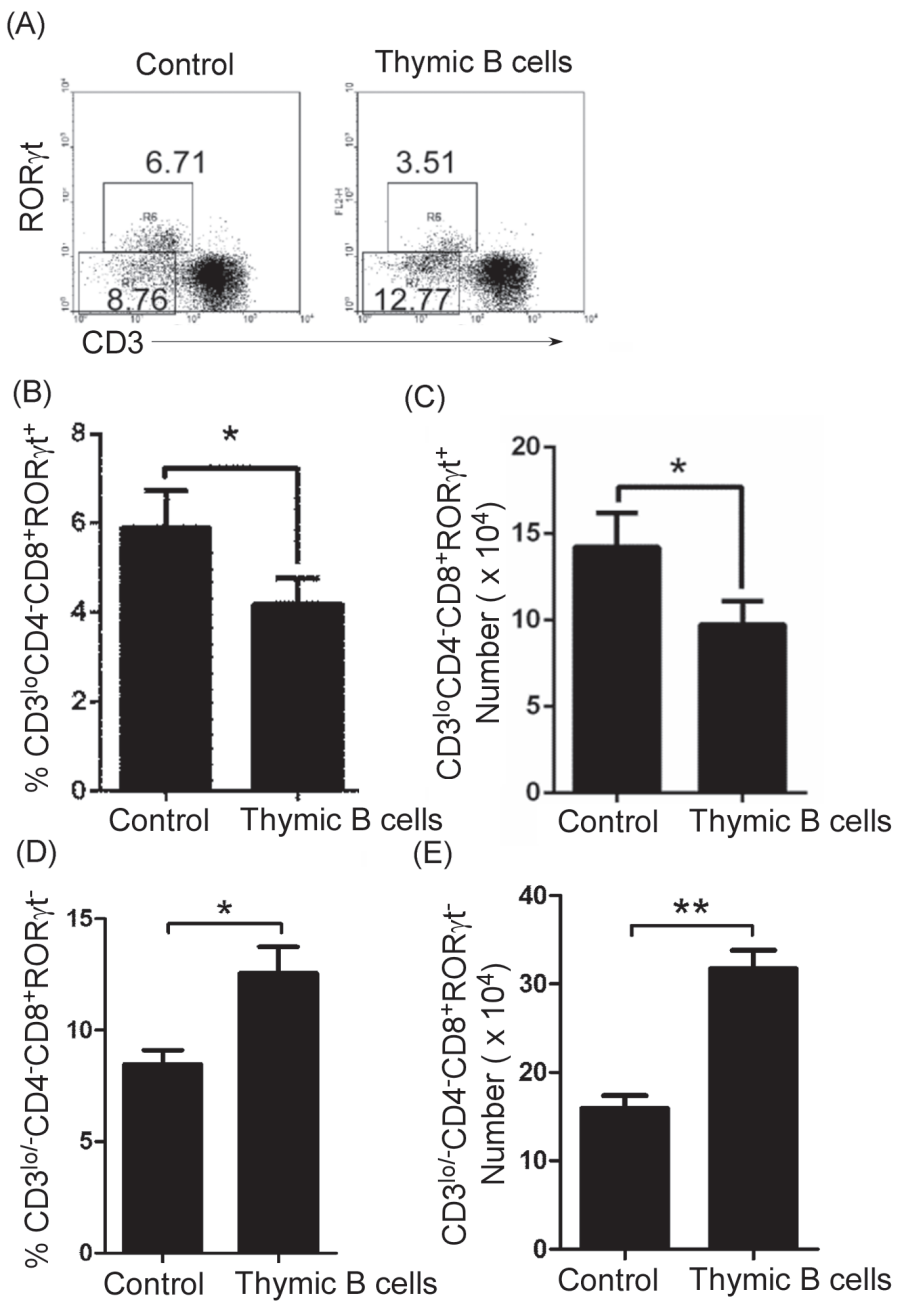
Thymic B-cell transfer up-regulated thymic CD4^-^CD8^+^CD3^lo/-^RORγt^-^ ISP T cells and reduced immature CD4^-^CD8^+^CD3loRORγt^+^T cells Thymic B cells from 7-9-week-old lupus-prone mice were sorted by B220 microbeads. 1 × 10^6^ cells per mouse were transferred into 7-9-week-old non-lupus-prone MRL/+ mice. The mice without B-cell transfer were used as the control. On day 21 after cell transfer, thymocytes were stained with anti-mouse CD4, CD8, CD3 and RORγt antibodies, and analyzed by FACS. The percentage **A.**, and the statistical results for the percentage **B.**, **D.**, and the absolute numbers **C.**, **E.**, of thymic CD4^-^CD8^+^CD3^lo/-^RORγt^-^ ISP **D.**, **E.**, and CD4^-^CD8^+^CD3^lo^RORγt^+^ **B.**, **C.**, T cells are shown. **B.**-**E.** Data are shown as mean + SEM (*n* = 18) from three independent experiments. *P < 0.05, **P < 0.01. Two tailed student's *t*-test. Error bars, s.e.m.

To further prove the effect of thymic B cells on ISP CD8^+^T-cell differentiation, we transferred thymic B cells from non-lupus-prone and lupus-prone mice into non-lupus-prone mice. We found that thymic B cells increased in mice that received B cells from non-lupus-prone and lupus-prone mice (Supplementary Figure S5A-S5C). Importantly, on day 21 after cell transfer the number of thymic B cells was higher in mice that received B cells from lupus-prone mice than in mice that received B cells from non-lupus-prone mice (Supplementary Figure S5A-S5C). These data suggest that thymic B cells from lupus-prone mice may expand. As expected, we found that thymic B-cells from both non-lupus-prone and lupus-prone mice up-regulated thymic CD4^-^CD8^+^CD3^lo/-^RORγt^-^ ISP T cells (Supplementary Figure S5, Figure 6A and 6D-6E). Critically, the extent of this increase in ISP cells is in line with the increase in thymic B-cell number (Supplementary Figure S5). These data suggest that thymic B cells from lupus-prone mice could block thymic ISP CD8^+^T-cell differentiation by B-cell expansion.

### IgG antibody controlled ISP CD8^+^T cell differentiation in lupus-prone mice

**Figure 7:**
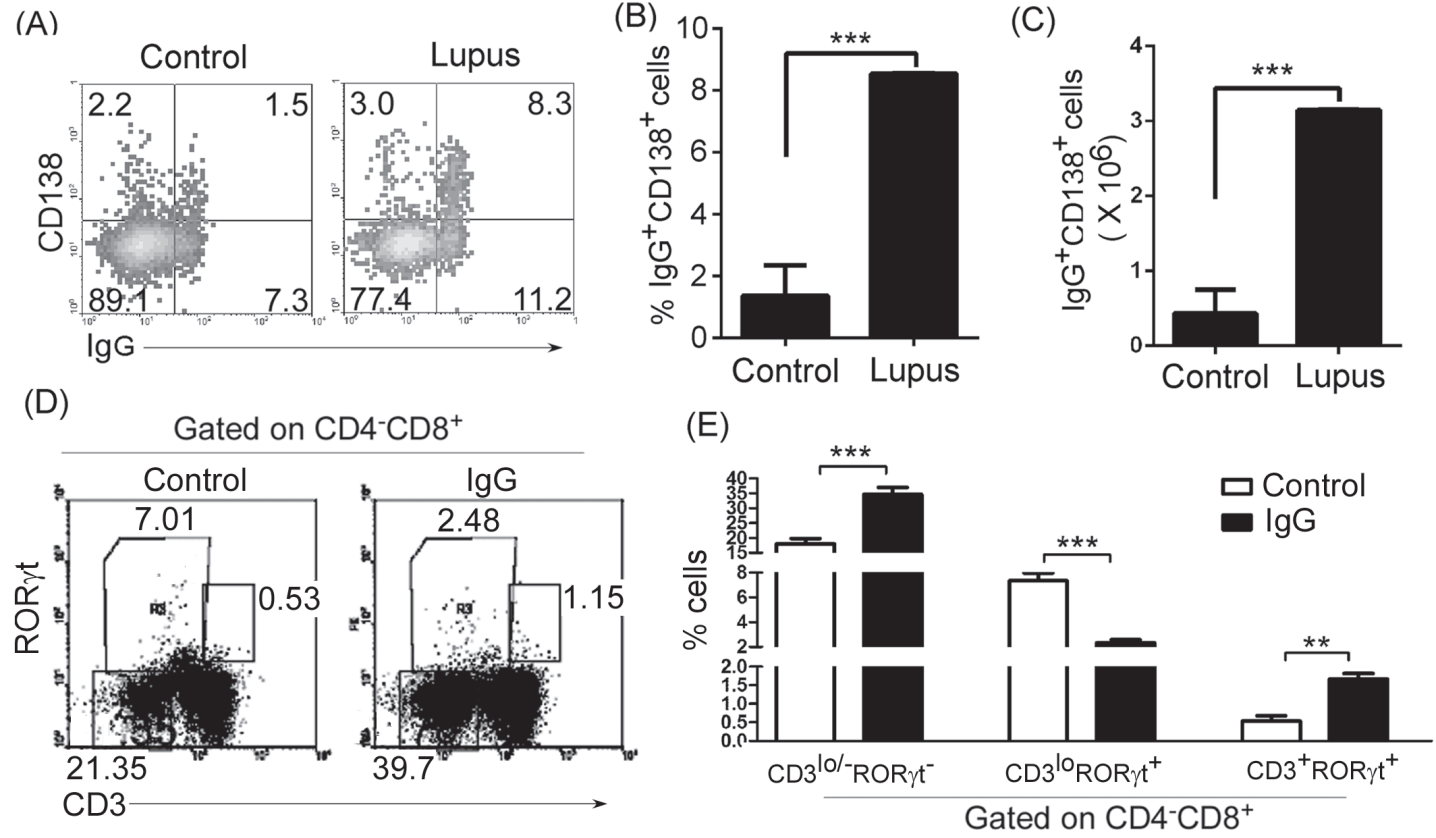
IgG up-regulated thymic CD4^-^CD8^+^CD3^lo/-^RORγt- ISP T cells, reduced immature CD4^-^CD8^+^CD3^lo^RORγt^+^T cells, and up-regulated mature CD4^-^CD8^+^CD3^+^RORγt^+^T cells in lupus-prone mice **A.**, **B.**, **C.** IgG-secreting CD138^+^ plasma cells increased in the thymus of lupus-prone mice. Thymocytes were isolated from 7-9-week-old non-lupus-prone MRL/+ (Control) and lupus-prone MRL/lpr (Lupus) mice (6 mice per group). Thymocytes were surface stained with anti-mouse B220 and CD138 and stained intracellularly with anti-mouse IgG antibody, and analyzed by flow cytometry (FACS). The percentage **A.**, the statistical results for the percentage **B.**, and the absolute numbers **C.**, of thymic IgG^+^CD138^+^ plasma cells on gated B220^lo/+^B cells are shown. **D.**, **E.** IgG up-regulated thymic CD4^-^CD8^+^CD3^lo/-^RORγt^-^ ISP T cells, reduced immature CD4^-^CD8^+^CD3^lo^RORγt^+^T cells, and up-regulated mature CD4^-^CD8^+^CD3^+^RORγt^+^T cells. IgG from 6-7-month-old lupus-prone mice was purified by affinity chromatography. 100 μg IgG per mouse was i.v. injected into 7-9-week-old none-lupus-prone mice. PBS was used as the control. On day 21 after IgG injection, thymocytes were stained with anti-mouse CD4, CD8, CD3 and RORγt antibodies, and analyzed by FACS. The percentage **D.**, and the statistical results for the percentage **E.**, of thymic CD4^-^CD8^+^CD3^lo/-^RORγt^-^ and CD4^-^CD8^+^CD3^lo^RORγt^+^ and CD4^-^CD8^+^CD3^+^RORγt^+^ T cells are shown. **B.**, **C.**, **E.** Data are shown as mean + SEM (*n* = 18) from three independent experiments. *P < 0.05, **P < 0.01, ***P < 0.001. **B.**, **C.** Two tailed student's *t*-test. **E.** Two-Way ANOVA plus Bonferroni post-tests were used to compare each column *vs* control column. Error bars, s.e.m.

To explore the mechanisms by which thymic B cells block thymic ISP CD8^+^T-cell differentiation in lupus-prone mice, we used affymetrix microarrays to examine the transcripts in thymic CD4^-^CD8^+^CD3^lo/-^ISP CD8^+^T-cells and CD4^-^CD8^+^CD3^+^T cells. We found that Fcgr2b (Fc receptor, IgG, low affinity IIb) and Fcer2a (Fc receptor, IgE, low affinity II, alpha polypeptide) were higher in CD4^-^CD8^+^CD3^lo/-^ISP CD8^+^T-cells than in CD4^-^CD8^+^CD3^+^T cells (Table I). Thus, we propose that IgG or IgE may affect ISP CD8^+^T cell differentiation. To prove this, we first determined the level of IgG-secreting plasma cells and found that IgG-secreting CD138^+^ plasma cells increased in the thymus of lupus-prone mice (Figure 7A-7C). These results suggest that thymic B cells control immature single positive (ISP) CD8^+^T cell differentiation by secreting IgG in lupus-prone mice. To further prove the proposal, polyclonal IgG antibodies were purified from C57BL/6, non-lupus-prone and lupus-prone mice, and i.v. injected into non-lupus prone mice. As expected, we found that IgG from C57BL/6, non-lupus-prone and lupus-prone mice up-regulated thymic CD4^-^CD8^+^CD3^lo/-^RORγt^-^ ISP T cells (Supplementary Figure 6, Figure 7D and 7E). Together, our data suggest that IgG, secreted by a higher level of plasma cells, could block thymic ISP CD8^+^T-cell differentiation in lupus-prone mice.

### Thymic B cells promoted the production of mature CD4^-^CD8+CD3^+^RORγt^+^T cells in lupus-prone mice

Apart from ISP CD8^+^T (CD4^-^CD8^+^CD3^lo/-^RORγt^-^) cells, thymic B cells may regulate the other two populations of cells (immature CD4^-^CD8^+^CD3^lo^RORγt^+^T and mature CD4^-^CD8^+^CD3^+^RORγt^+^T) (Figure 4A and Supplementary Figure 4A). First, we detected the level of two populations of cells in lupus-prone mice. As expected, we found that immature CD4^-^CD8^+^CD3^lo^RORγt^+^T cells decreased, whereas mature CD4^-^CD8^+^CD3^+^RORγt^+^T cells increased in lupus-prone mice (Figure 4C, and 4F-4I). These results suggest that lupus induces thymic immature CD4^-^CD8^+^CD3^lo^RORγt^+^T cells to differentiate into mature CD4^-^CD8^+^CD3^+^RORγt^+^T cells.

To assess the ability of thymic B cells to transform thymic immature CD4^-^CD8^+^CD3^lo^RORγt^+^T cells into mature CD4^-^CD8^+^CD3^+^RORγt^+^T cells, we first analyzed these cells in WT and CD19^cre^ mice. Our data demonstrated that thymic mature CD4^-^CD8^+^CD3^+^RORγt^+^T cells decreased and thymic immature CD4^-^CD8^+^CD3^lo^RORγt^+^T cells increased in CD19^cre^ mice (Supplementary Figure S1C and S1D). These results suggest that thymic B cells may promote thymic immature CD4^-^CD8^+^CD3^lo^RORγt^+^T cells to differentiate into mature CD4^-^CD8^+^CD3^+^RORγt^+^T cells.

To assess the effect of thymic B cells on the transformation of thymic immature CD4^-^CD8^+^CD3^lo^RORγt^+^T cells into mature CD4^-^CD8^+^CD3^+^RORγt^+^T cells in autoimmune diseases, we injected lupus-inducing pristane into homozygous CD19^cre^ mice. In accordance with the data in lupus-prone mice, lupus-inducing pristane reduced thymic immature CD4^-^CD8^+^CD3^lo^RORγt^+^T cells and up-regulated mature CD4^-^CD8^+^CD3^+^RORγt^+^T cells (Figure 5A and 5B). Critically, we found that in homozygous CD19^cre^ mice, lupus-inducing pristane did not reduce thymic immature CD4^-^CD8^+^CD3^lo^RORγt^+^T cells and up-regulated mature CD4^-^CD8^+^CD3^+^RORγt^+^T cells (Figure 5A and 5B). In accordance with these results, we found that B-cell reduction with TACI-IgG reduced mature thymic CD4^-^CD8^+^CD3^+^RORγt^+^T cells and up-regulated immature thymic CD4^-^CD8^+^CD3^lo^RORγt^+^T cells in lupus-prone MRL/lpr mice (Supplementary Figure S2F and S2G). These data suggest that thymic B cells promote thymic immature CD4^-^CD8^+^CD3^lo^RORγt^+^T cells to differentiate into mature CD4^-^CD8^+^CD3^+^RORγt^+^T cells in lupus-induced or -prone mice.

To further prove the effect of thymic B cells on the differentiation of thymic immature CD4^-^CD8^+^CD3^lo^RORγt^+^T cells into mature CD4^-^CD8^+^CD3^+^RORγt^+^T cells, we transferred thymic B cells from non-lupus-prone and lupus-prone mice into non-lupus-prone mice. Importantly, we found that thymic B-cells reduced thymic immature CD4^-^CD8^+^CD3^lo^RORγt^+^T cells and up-regulated mature CD4^-^CD8^+^CD3^+^RORγt^+^T cells cells (Supplementary Figure S5D-S5F, and Figure 6A-6C). Critically, the extent of the decrease in thymic immature CD4^-^CD8^+^CD3^lo^RORγt^+^T cells and increase in mature CD4^-^CD8^+^CD3^+^RORγt^+^T cells cells is in line with the increase in the thymic B-cell number (Supplementary Figure S5). These data suggest that B cells may be involved in the differentiation of immature thymic CD4^-^CD8^+^CD3^lo^RORγt^+^T cells into mature CD4^-^CD8^+^CD3^+^RORγt^+^T cells induced by thymic B-cell expansion in lupus-prone mice.

### IgG antibody induced the differentiation of thymic immature CD4^-^CD8^+^CD3loRORγt^+^T cells into mature CD4^-^CD8^+^CD3^+^RORγt^+^T cells

IgG-secreting CD138^+^ plasma cells increased in the thymus of lupus-prone mice (Figure 7A-7C). Thus, we proposed that IgG may be involved in the differentiation of immature thymic CD4^-^CD8^+^CD3^lo^RORγt^+^T cells into mature CD4^-^CD8^+^CD3^+^RORγt^+^T cells in lupus-prone mice. As expected, we found that IgG from C57BL/6, non-lupus-prone and lupus-prone mice reduced thymic immature CD4^-^CD8^+^CD3^lo^RORγt^+^T cells and up-regulated mature CD4^-^CD8^+^CD3^+^RORγt^+^T cells (Supplementary Figure S6, Figure 7D and 7E). Thus, our data suggest that IgG antibody, secreted by a higher level of plasma cells, could induce the differentiation of immature thymic CD4^-^CD8^+^CD3^lo^RORγt^+^T cells into mature CD4^-^CD8^+^CD3^+^RORγt^+^T cells in lupus-prone mice.

## DISCUSSION

SLE is a relapsing and remitting, chronic autoimmune inflammatory disease characterized by the production of a wide array of autoantibodies. Both antibodies and autoreactive T cells play significant roles in its pathogenesis [10]. T cells are recognized as crucial in the pathogenicity of SLE through their capacity to communicate with and offer enormous help to B cells in driving autoantibody production [29]. We demonstrated here that IgG, secreted by thymic B cells up-regulated by lupus, blocked the differentiation from thymic CD8^+^ISP cells and induced the differentiation of novel immature CD4^-^CD8^+^CD3^lo^RORγt^+^T cells into mature RORγt^+^CD8^+^ T cells. The study suggests that B cells from lupus could regulate thymic T cell development.

CD8^+^ISP cells represent a stage of rapid proliferation, driven by signals emanating from pre-TCR. The resultant DP cells comprise 85%-90% of an adult thymus [17]. Previous studies have shown that a block in T cell development at the ISP to DP transition results in a significant increase in CD8^+^ ISP cells in transgenic mice overexpressing the Id2 HLH protein display [30] and disruption of Tcf-1 in Tcf (VII) mutant mice [31]. We demonstrated here that IgG, secreted by thymic B cells up-regulated by lupus, blocked the differentiation of thymic CD8^+^ISP to DP cells with a significant increase in CD8^+^ ISP cells (Figure 4-7, Supplementary Figure S2, S5 and S6).

A previous study demonstrated an increase in mature CD4^+^ and CD8^+^ SP in E2A (E12/E47)-mutant mice with a partial block at the DN1 and ISP stages [17]. In accordance with this study, we found that thymic CD4^+^CD8^-^ and CD4^-^CD8^+^T cell percentage and absolute numbers increased in lupus-prone mice (Figure 1D-1F, Figure 2D, 2E, Supplementary Figure S2D and S2E) with a significant increase in CD8^+^ ISP cells (Figure 4C-4E, Figure 5A, 5B, Supplementary Figure S2F, S2G). These studies suggest that a block in T cell development at the ISP to DP transition may be beneficial for a significant increase in mature CD4^+^ and CD8^+^ SP cells.

The CD4 or CD8 lineage ‘decision’ may be matched to MHC specificity. MHC I and II-restricted thymocytes become cytotoxic CD8 cells and helper CD4 cells, respectively [32]. The last several years have seen connections emerging between transcription factors involved in the choice of the CD4 or CD8 lineage. Two transcription factors, Thpok and Runx3, specifically expressed in CD4 and CD8 differentiating thymocytes, respectively, are important for this process [33–36]. Thpok is required for CD4 commitment and acts at least in part by repressing the expression of CD8 lineage genes, including Runx3 [33, 37–39]. Runx3 is important for the silencing of CD4 in CD8 cells [34, 36], and the complete disruption of Runx activity (Runx3 and the partly redundant factor Runx1) prevents CD8 cell development [37, 40]. Our data demonstrated that thymic B cells mainly regulated thymic CD4^-^CD8^+^ but not CD4^+^CD8^-^ T cells in lupus-induced or -prone mice (Figure 2D, 2E, Supplementary Figure S2D and S2E). Altogether, these results suggest that thymic B cells may regulate the CD4 or CD8 lineage ‘decision’. Thus, it is possible that B-cell reduction results in a reduction of thymic CD4^-^CD8^+^ T cells and an increase of CD4^+^CD8^-^ T cells in pristane-treated mice (Figure 2D and 2E). The mechanisms by which thymic B cells regulate the CD4 or CD8 lineage ‘decision’ are worthy of further exploration.

Many studies have shown that the CD8^+^/CD4^+^T cell ratio [14] and Tc17 cells are increased in SLE patients [15]. In line with these studies, our data suggest that the ratio of peripheral CD8^+^ to CD4^+^ T cells and RORγt^+^CD8^+^ T cells increased in lupus-prone (Figure 3) and pristane-treated (Supplementary Figure S3) mice. In accordance with the increased RORγt^+^CD8^+^ T cells, IL-17-producing Tc17 increased in prostane-treated mice (Supplementary Figure S3E and S3F). Tc17 are effector cells that promote inflammation, adaptive immunity and autoimmunity by producing IL17 and other inflammatory cytokines [27]. Thus, thymic B cells may affect autoimmunity partly by regulating the thymic CD4 or CD8 lineage ‘decision’ to control the ratio of peripheral CD8^+^ to CD4^+^ T cells and IL-17-producing T cells.

Consistent with peripheral data, we found that thymic mature CD4^-^CD8^+^CD3^+^RORγt^+^ T increased in lupus-prone mice (Figure 4-7, Supplementary Figure S2, S5 and S6). Importantly, CD4^-^CD8^+^CD3^lo^RORγt^+^ T cells were induced from a novel population of thymic immature T cells (CD4^-^CD8^+^CD3^lo^RORγt^+^) by IgG secreted by B cells from lupus-prone mice (Figure 4-7, Supplementary Figure S2, S5 and S6).

In conclusion, B cells blocked the differentiation from thymic CD8^+^ISP and induced the differentiation of novel immature CD4^-^CD8^+^CD3^lo^RORγt^+^T cells into mature RORγt^+^CD8^+^ T cells by producing IgG antibody in lupus-prone mice. The study suggest that lupus B cells may up-regulate peripheral pathogenic CD8^+^T cells by regulating thymic immature and mature CD8^+^T cells. Infiltrating CD8^+^ T cells and antibody in the lupus kidney have the potential to mediate injury.

## MATERIALS AND METHODS

### Mice

Seven-to-nine-week-old C57BL/6 mice (Huafukang Corp., Beijing, China), 7-9-week-old homozygous CD19^cre^ on background of C57BL/6 mice, 7-9-week-old or 6-month-old female lupus-prone MRL/MpJ/lpr/lpr (MRL/lpr) mice and age- and sex-matched MRL/MpJ/+/+ (MRL/+) (Nanjing Biomedical Research Institute of Nanjing University, Nanjing, China) were bred in our animal facilities under specific pathogen-free conditions. Care, use, and treatment of mice in this study were in strict agreement with international guidelines for the care and use of laboratory animals. This study was approved by the Animal Ethics Committee of the Beijing Institute of Basic Medical Sciences.

### Cytometric analysis and intracellular cytokine staining

All cell experiments were strictly prepared on ice, unless otherwise stated in other specific procedures. Cells (1×10^6^ cells/sample) were washed with fluorescence-activated cell sorting staining buffer (phosphate-buffered saline, 2% fetal bovine serum or 1% bovine serum albumin, 0.1% sodium azide). All samples were incubated with anti-Fc receptor Ab (clone 2.4G2, BD Biosciences, San Jose, CA), prior to incubation with other Abs diluted in fluorescence-activated cell sorting buffer supplemented with 2% anti-Fc receptor Ab. For intracellular cytokine staining, cells were collected and fixed for 50 min with 1mL fixation buffer (IC Fixation and Permeabilization kit, eBioscience, San Diego, CA). After washing, the fixed cells were stained. The samples were filtered immediately before analysis or cell sorting to remove any clumps. The following antibodies were used: fluorescence-conjugated anti-mouse B220 (eBioscience, RA3-6B2), CD3 (eBioscience, 145-2C11), CD4 (eBioscience, GK1.5), CD8 (Biolegend, 53-6.7), RORγt (eBioscience, B2D), IL-17A (eBioscience, eBio17B7), CD24 (eBioscience, M1/69), CD138 (Biolegend, 281-2), and IgG (Santa Cruz Biotech, F1306) antibodies. Data collection and analyses were performed on a FACS Calibur flow cytometer using CellQuest software.

### 2,6,10,14-Tetramethylpentadecane (pristane) were *i.v.* injected into mice

Experimental lupus induced by the hydrocarbon oil 2,6,10,14-Tetramethylpentadecane (TMPD; also known as pristane) displays many key immunological and clinical features of human SLE [41]. Treatment of mice with pristane were performed as described [25]. 0.5 ml pristane (Cat no. 138462500, Fisher Scientific Corp. USA) per mice were injected i.p. into 7-9-week-old female or male C57BL/6 or homozygous CD19^cre^ mice. On day 21 after injection, Single-cell suspension of thymocytes was got simply by mechanical disruption, and analyzed by FACS.

### Cell sorting

B cells from the thymus were sorted using B220 microbeads. Based on the staining with fluorescence-conjugated anti-mouse CD4, CD8 and CD3 antibodies, thymic CD4^-^CD8^+^CD3^lo/-^ and CD4^-^CD8^+^CD3^+^ T cells was sorted by multicolor flow cytometry. All flow cytometry data were acquired with FACSCanto, FACSCantoII, or FACSAria (BD Biosciences). Live lymphocyte-sized cells for T- and FlowJo software (Tree Star, Ashland, OR). The purity of sorted cells was shown to be > 95% by flow cytometric analysis.

### Adoptive transfer of Thymic B cells

Thymocytes were separated as described [24]. Single-cell suspension of thymocytes from 7-9-week-old lupus-prone MRL/lpr (Lupus) mice was got simply by mechanical disruption. Thymic B cells were sorted by B220 microbeads (AutoMACS, Miltenyi Biotic). 1 × 10^6^ thymic B cells per mouse were adoptively transferred *in vein* (i.v.) into 7-9-week-old non-lupus-prone MRL/+ mice. Non-B-cell transfer group was used as the control.

### Treatment of non-lupus-prone MRL/+ mice with IgG

Protein A/G is a recombinant protein expressed in Escherichia coli (50.5 kDa; 6 IgG binding sites) and is recommended for purifying polyclonal IgG from many species. Polyclonal mouse IgG from the serum of 7-9-week-old C57BL/6, non-lupus-prone MRL/+ mice and lupus-prone MRL/lpr mice or 6-7-month-old lupus-prone MRL/lpr mice was purified by Pierce Recombinant Protein A/G (Thermo Scientific Cat #21186). 100 μg IgG per mouse were i.v. injected into 7-9-week-old non-lupus-prone mice. PBS was used as the control.

### Affymetrix microarrays

Affymetrix microarrays were performed as described [42]. Total RNA was extracted from thymic CD4^-^CD8^+^CD3^lo/-^ and CD4^-^CD8^+^CD3^+^ T cells with Trizol and purified over Qiagen RNeasy columns (Qiagen). Synthesis and labeling of RNA and hybridization of arrays was conducted. Stained arrays (430 2.0) were scanned on an Agilent Gene Array Scanner (Affymetrix). Most significantly changed mRNA transcripts are shown.

### Statistics

Statistics were analyzed by using GraphPad Prism (version 5.0, GraphPad Software Inc., USA). The data were shown as mean ± standard error of the mean (SEM). Student's t test was employed to determine significance between two groups (paired or unpaired) and One-Way or Two-Way ANOVA analysis was used to determine significance among several groups. Differences were considered statistically significant when *p* < 0.05.

## SUPPLEMENTARY MATERIALS FIGURES AND TABLES



## Abbreviations

ISP: immature single positive
SLE: systemic lupus erythematosus
FACS: flow cytometry
Pristane: 2,6,10,14-Tetramethylpentadecane
WT: wild type
LN: lymph nodes
