# Supplementary material for: B cells regulate thymic CD8+T cell differentiation in lupus-prone mice
Source: Oncotarget. 2017 Jul 5;8(52):89486–99. doi: 10.18632/oncotarget.19002 (PMC5685686; doi:10.18632/oncotarget.19002)
Supplement: Supplementary file 1 [file oncotarget-08-89486-s001.pdf]

B cells regulate thymic CD8^{+}T cell differentiation in lupus-prone mice

Supplementary Material

Supplementary Figure 1

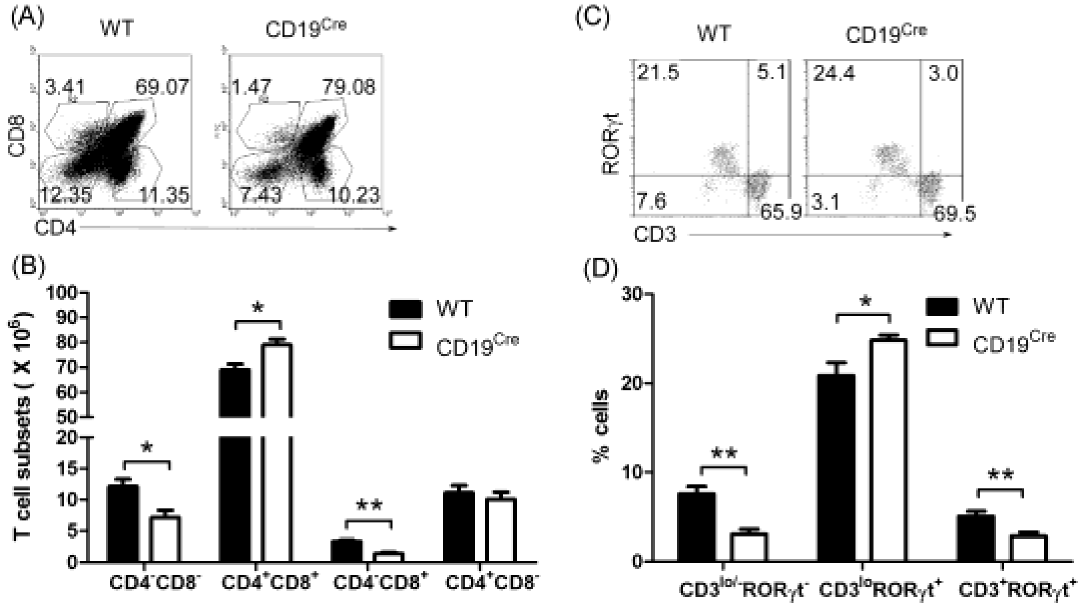

Supplementary Figure S1: Thymic CD4^{-}CD8^{+}T cells, CD4^{-}CD8^{+}CD3$^{lo/-}$RORγt^{-} ISP T cells and mature CD4^{-}CD8^{+}CD3^{+}RORγt^{+}T cells decreased and immature CD4^{-}CD8^{+}CD3^{lo}RORγt^{+}T cells increased in CD19^{Cre} mice. Single-cell suspension of thymocytes from 7-9-week-old wild type (WT) C57BL/6 mice and homozygous CD19^{cre} mice on background of C57BL/6 mice (6 mice per group) was got simply by mechanical disruption. **A., B.** Thymic CD4^{-}CD8^{+}T cells decreased in CD19^{Cre} mice. Thymocytes were stained with anti-mouse CD4 and CD8 antibodies, and analyzed by FACS. The percentage **A.**, and the absolute numbers **B.**, of thymic CD4^{-}CD8^{-} and CD4^{+}CD8^{+}T, CD4^{+}CD8^{-} and CD4^{-}CD8^{+}T cells are shown. **C., D.** Thymic CD4^{-}CD8^{+}CD3$^{lo/-}$RORγt^{-} ISP T cells and mature CD4^{-}CD8^{+}CD3^{+}RORγt^{+}T cells decreased and immature CD4^{-}CD8^{+}CD3^{lo}RORγt^{+}T cells increased in CD19^{Cre} mice. Thymocytes were stained with anti-mouse CD4, CD8, CD3 and RORγt antibodies, and analyzed by FACS. The percentage **C.**, and the statistical results for the percentage **D.**, of thymic CD3$^{lo/-}$RORγt^{-} ISP, CD3^{lo}RORγt^{+} and CD3^{+}RORγt^{+} T cells on gated of CD4^{-}CD8^{+}T cells are shown. Data are shown as mean + SEM (n=18) from three independent

experiments. *$P < 0.05$, **$P < 0.01$. Two-Way ANOVA plus Bonferroni post-tests to compare each column vs WT column. Error bars, s.e.m.

Supplementary Figure 2

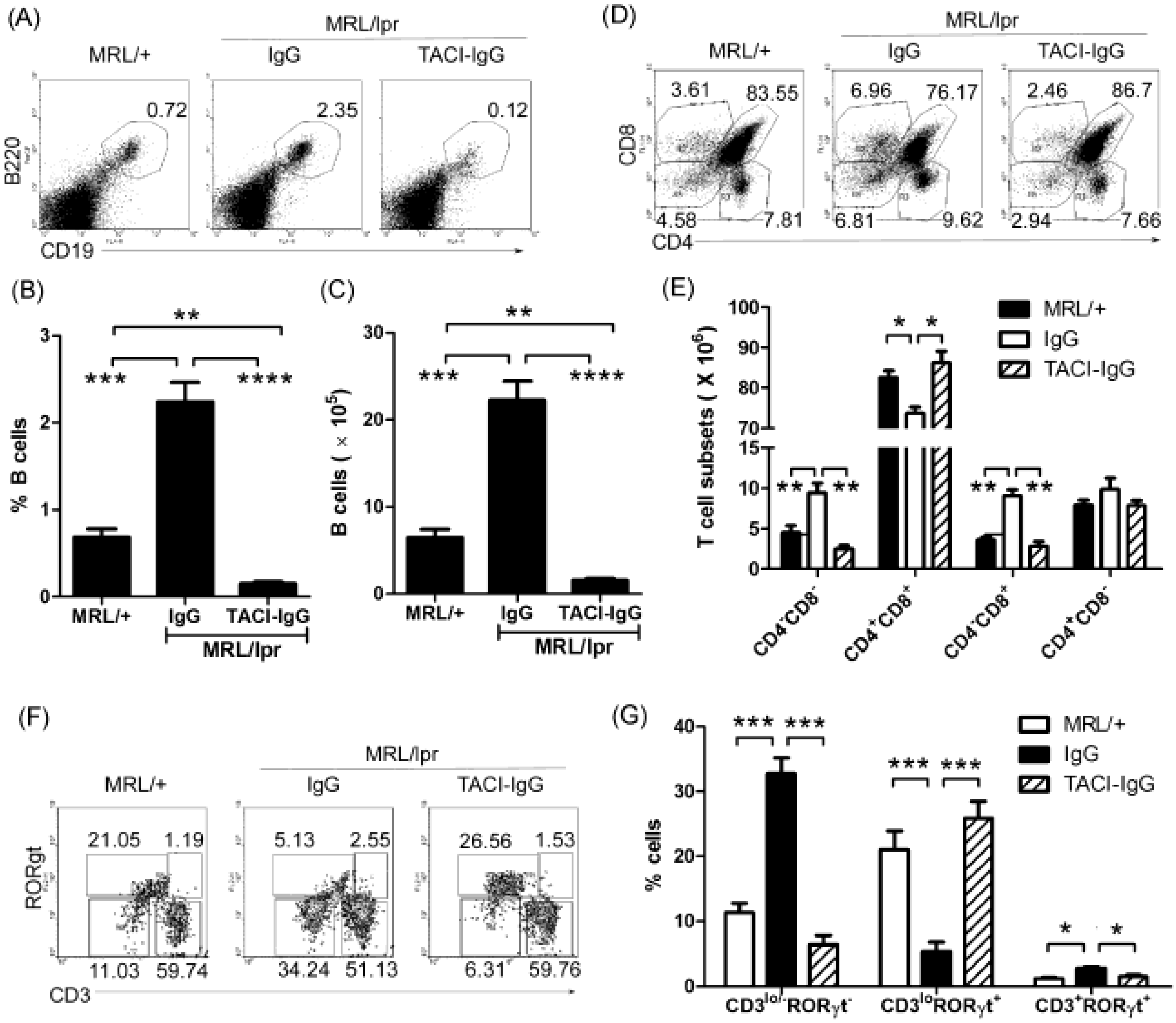

Supplementary Figure S2: B-cell depletion reduced thymic CD4$^-$CD8$^+$T cells, CD4$^-$CD8$^+$CD3$^{lo/-}$RORγt$^-$ ISP T cells and mature CD4$^-$CD8$^+$CD3$^+$RORγt$^+$T cells and up-regulated immature CD4$^-$CD8$^+$CD3^{lo}RORγt$^+$T cells in lupus-prone MRL/lpr mice. Lupus-prone MRL/lpr mice (6 mice per group) were injected i.p. with 5 mg/kg TACI-IgG or IgG control at 1, 2, 3, and 4 weeks (two times per week) after the mice reached 9-week-old. Age- and sex-matched non-lupus-prone MRL/+ mice were used as the control. On day 4-6 after treatment, thymocytes were collected. **A., B., C.** Thymic B cells decreased in TACI-IgG-treated MRL/lpr mice. Thymocytes were stained with anti-mouse B220 and CD19 antibody and analyzed by FACS. The percentage **A.,** the statistical results for the percentage **B.,** and the absolute numbers **C.,** of thymic B cells

are shown. **D., E.** Thymic CD4$^-$CD8$^+$T cells but not CD4$^+$CD8$^-$T cells decreased in B cells-reduced mice. Thymocytes were stained with anti-mouse CD4 and CD8 antibodies, and analyzed by FACS. The percentage **D.**, and the absolute numbers **E.**, of thymic CD4$^-$CD8$^-$ and CD4$^+$CD8$^+$T, CD4$^+$CD8$^-$ and CD4$^-$CD8$^+$T cells are shown. **F., G.** B-cell depletion reduced thymic CD4$^-$CD8$^+$CD3$^{lo/-}$RORγt$^-$ ISP T cells and mature CD4$^-$CD8$^+$CD3$^+$RORγt$^+$T cells and up-regulated immature CD4$^-$CD8$^+$CD3^{lo}RORγt$^+$T cells. Thymocytes were stained with anti-mouse CD4, CD8, CD3 and RORγt antibodies, and analyzed by FACS. The percentage **F.**, and the statistical results for the percentage **G.**, of thymic CD3$^{lo/-}$RORγt$^-$ ISP, CD3^{lo}RORγt$^+$ and CD3$^+$RORγt$^+$ T cells on gated of CD4$^-$CD8$^+$T cells are shown. Data are shown as mean + SEM (n=18) from three independent experiments. *$P < 0.05$, **$P < 0.01$, ***$P < 0.001$, ****$P < 0.0001$. One-Way **B., C.** and Two-Way **E., G.** ANOVA plus Bonferroni post-tests to compare each column vs control (Ig-treated group) column. Error bars, s.e.m.

Supplementary Figure 3

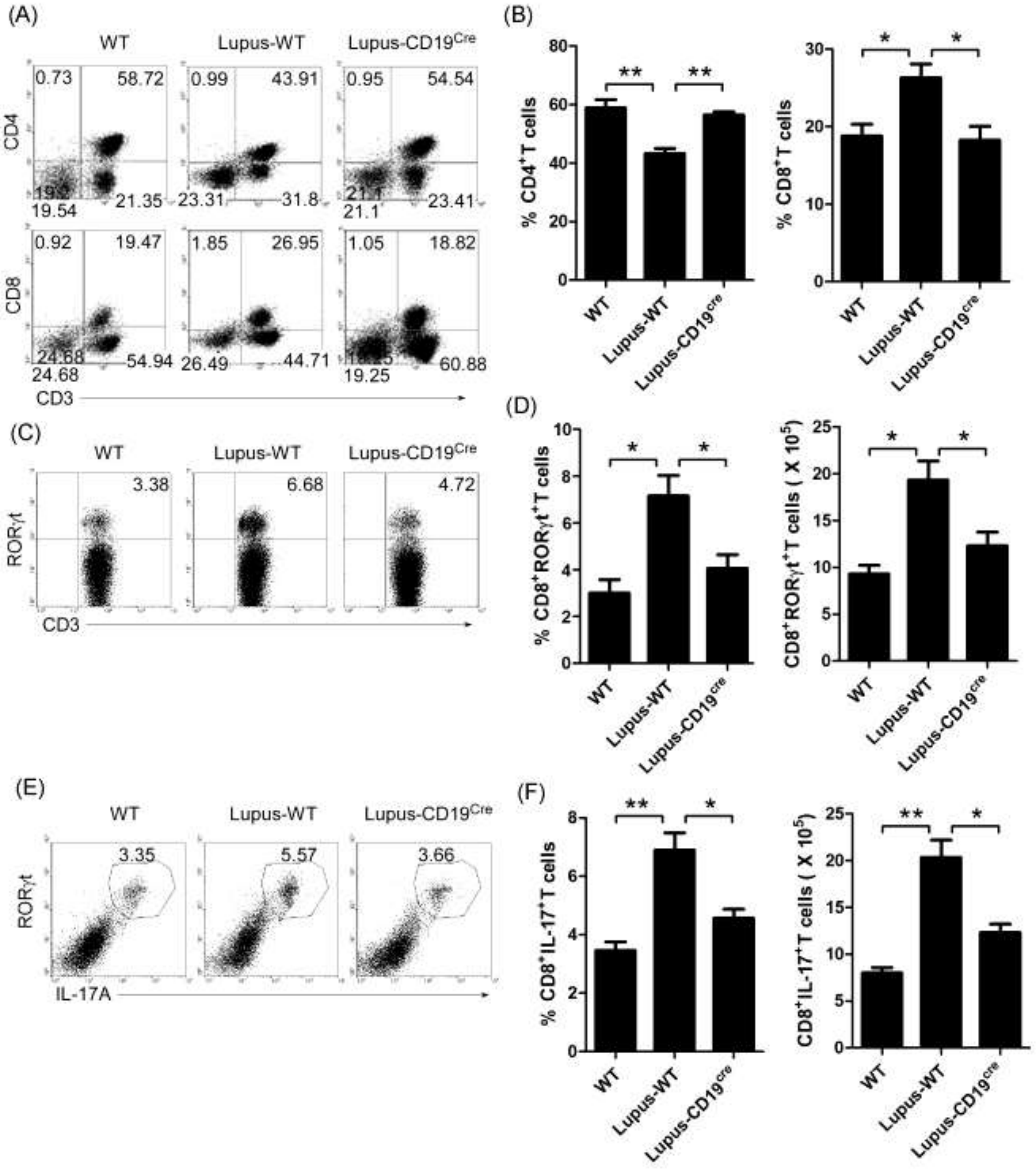

Supplementary Figure S3: B cells positively regulated peripheral mature CD8$^+$ and RORγt$^+$CD8$^+$ T cells in pristane-treated mice. Lymphocytes were separated from lymph nodes of WT, pristane-treated WT or CD19^{cre} mice described in Figure 2D (6 mice per group). **A., B.** B cells depletion reduced the ratio of peripheral CD8$^+$ to CD4$^+$ T cells in pristane-treated mice. Lymphocytes were stained with anti-mouse CD3,

CD4, and CD8 antibodies, and analyzed by FACS. **A.** Quadrants indicate percentage of $CD3^+$, $CD4^+$ and $CD8^+$ T cells. **B.** The statistical results for the percentage of $CD4^+$ and $CD8^+$ T cells are shown. **C., D.** B cells depletion reduced peripheral $ROR\gamma t^+CD8^+$ T cells in pristane-treated mice. Lymphocytes were stained with anti-mouse CD4, CD8, CD3, and RORγt antibodies, and analyzed by FACS. **C.** Quadrants indicate percentage of RORγt-expressing cells on gated of $CD4^-CD8^+$ T cells. **D.** The statistical results for the percentage (Left panel) and the absolute numbers (Right panel) of $CD4^-ROR\gamma t^+CD8^+$ T cells are shown. **E., F.** B cells depletion reduced peripheral IL-$17^+ROR\gamma t^+CD8^+$ T cells in pristane-treated mice. Lymphocytes were stained with anti-mouse CD4, CD8, IL-17A, and RORγt antibodies, and analyzed by FACS. **E.** Quadrants indicate percentage of IL-17A and RORγt-expressing cells on gated of $CD4^-CD8^+$ T cells. **F.** The statistical results for the percentage (Left panel) and the absolute numbers (Right panel) of IL-17-producing $CD4^-ROR\gamma t^+CD8^+$ T cells are shown. Data are shown as mean + SEM (n=24) from four independent experiments. One-Way ANOVA plus Bonferroni post-tests to compare each column vs pristane-treated column. Error bars, s.e.m. $^*P < 0.05$, $^{**}P < 0.01$.

Supplementary Figure 4

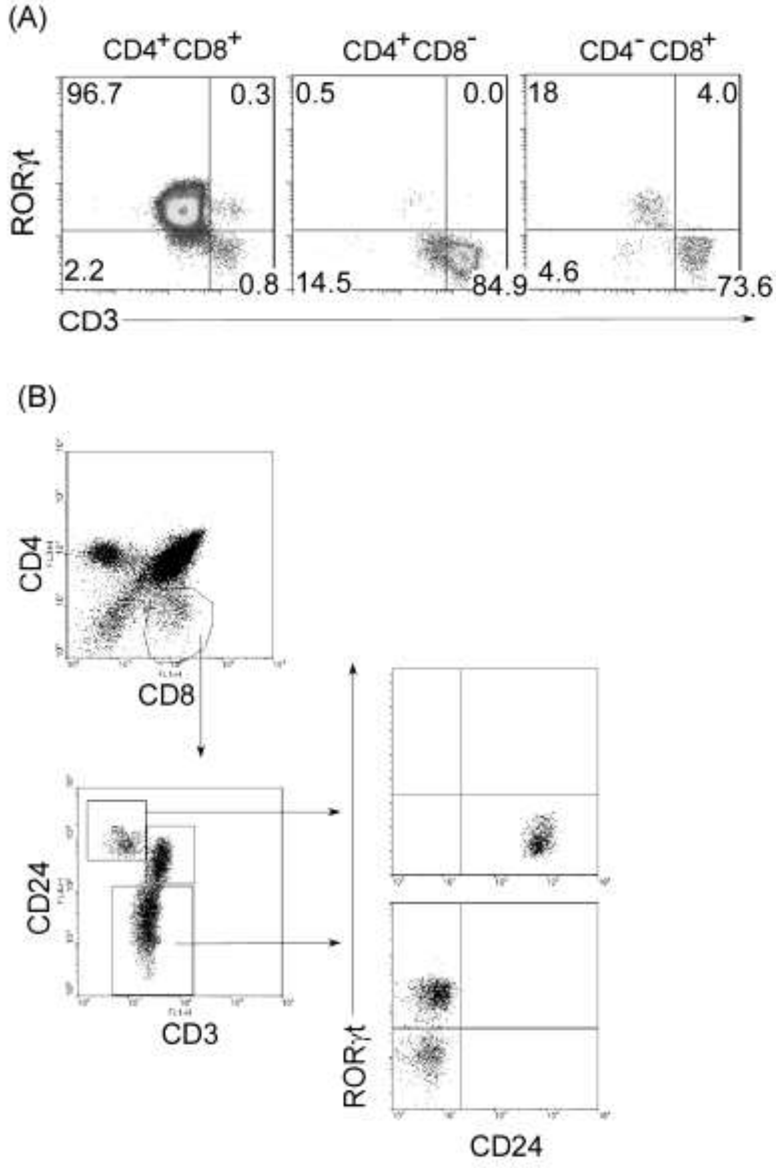

Supplementary Figure S4: Thymic ISP CD8^{+}T cells expressed high level of CD24 but not RORγt. Single-cell suspension of thymocytes from 7-9-week-old non-lupus-prone MRL/+ mice (6 mice) was got simply by mechanical disruption. Thymocytes were stained with anti-mouse CD4, CD8, CD3, CD24 and RORγt antibody and analyzed by flow cytometry (FACS). **A.** The percentage of RORγt- and/or CD3-expressing cells on gated of CD4^{+}CD8^{+}, CD4^{+}CD8^{-} and CD4^{-}CD8^{+} T cells. **B.** RORγt and CD24 expression (lower and right panel) in CD3^{lo}CD24^{hi} and CD3^{mi}CD24^{-} cells (lower and left panel) on gated of CD4^{-}CD8^{+} T cells (upper panel).

Supplementary Figure 5

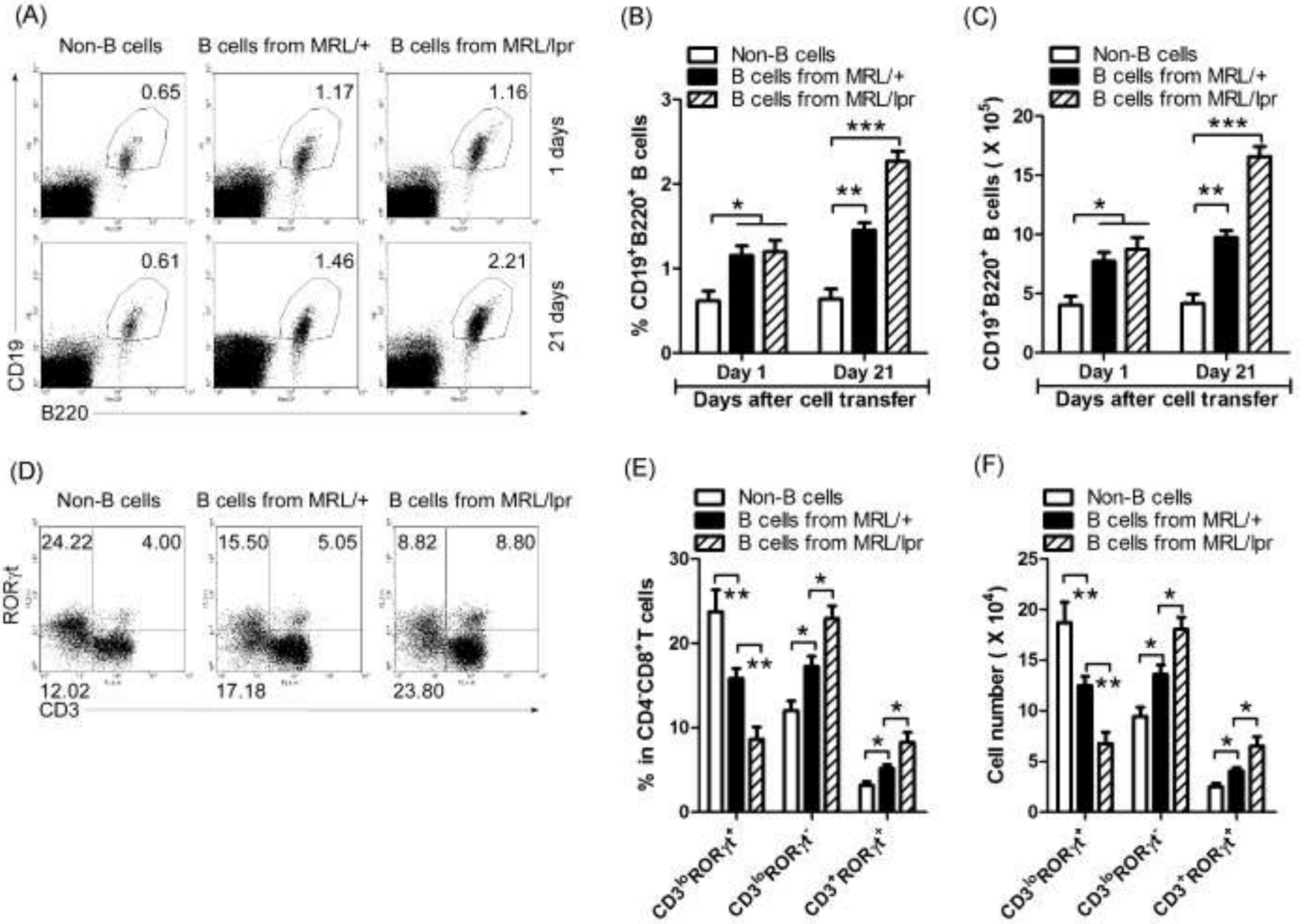

Supplementary Figure S5: Thymic B-cell transfer up-regulated thymic CD4$^-$CD8$^+$CD3$^{lo/-}$RORγt$^-$ ISP T cells and mature CD4$^-$CD8$^+$CD3$^+$RORγt$^+$T cells cells and reduced immature CD4$^-$CD8$^+$CD3^{lo}RORγt$^+$T cells. Thymic B cells from 7-9-week-old non-lupus-prone MRL/+ and lupus-prone MRL/lpr mice were stained by anti-mouse CD19 and B220 antibodies and sorted by FACS. 1 X 10^6 cells per mouse were transferred into 7-9-week-old non-lupus-prone MRL/+ mice. The mice without B-cell transfer were used as the control. **A., B., C.** Thymic B cells increased in thymic B-cell-transferred mice. On day 1 and 21 after cell transfer, thymocytes were stained with anti-mouse B220 and CD19, and analyzed by FACS. The percentage **A.**, and the statistical results for the percentage **B.**, and the absolute numbers **C.**, of thymic CD19$^+$B220$^+$B cells were shown. **D., E., F.** Thymic immature CD4$^-$CD8$^+$CD3^{lo}RORγt$^+$T cells decreased, whereas CD4$^-$CD8$^+$CD3$^{lo/-}$RORγt$^-$ ISP T cells and mature CD4$^-$CD8$^+$CD3$^+$RORγt$^+$T cells increased in thymic B-cell-transferred mice. On day 21 after

cell transfer, thymocytes were stained with anti-mouse CD4, CD8, CD3 and RORγt antibodies, and analyzed by FACS. The percentage **D.,** and the statistical results for the percentage **E.,** and the absolute numbers **F.,** of thymic $CD4^{-}CD8^{+}CD3^{lo}ROR\gamma t^{+}$, $CD4^{-}CD8^{+}CD3^{lo/-}ROR\gamma t^{-}$, and $CD4^{-}CD8^{+}CD3^{+}ROR\gamma t^{+}$ T cells are shown. Data are shown as mean + SEM (n=18) from three independent experiments. **P* < 0.05, ***P* < 0.01. Two-Way ANOVA plus Bonferroni post-tests to compare each column vs control column. Error bars, s.e.m.

Supplementary Figure 6

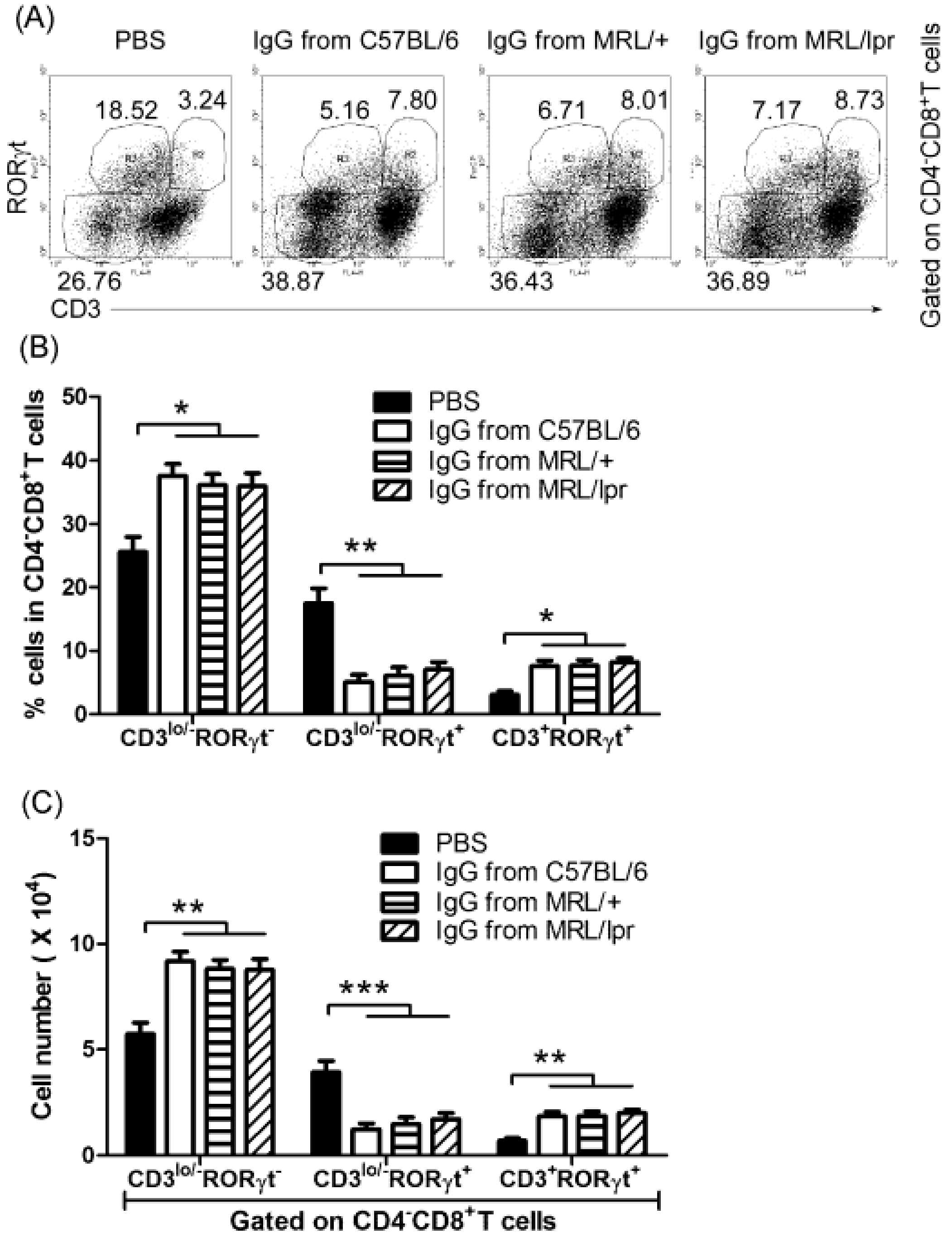

Supplementary Figure S6: IgG up-regulated thymic CD4$^-$CD8$^+$CD3$^{lo/-}$RORγt$^-$ ISP T cells, reduced immature CD4$^-$CD8$^+$CD3^{lo}RORγt$^+$T cells, and up-regulated mature CD4$^-$CD8$^+$CD3$^+$RORγt$^+$T cells. IgG from 7-9-week-old C57BL/6, non-lupus-prone MRL/+ mice and lupus-prone MRL/lpr mice was purified by affinity chromatography. 100 μg IgG per mouse were i.v. injected into 7-9-week-old none-lupus-prone MRL/+ mice. PBS was used as the control. On day 21 after IgG was

injected, thymocytes were stained with anti-mouse CD4, CD8, CD3 and RORγt antibodies, and analyzed by FACS. The percentage **A.,** the statistical results for the percentage **B.,** and the absolute numbers **C.,** of thymic $CD4^{-}CD8^{+}CD3^{lo/-}ROR\gamma t^{-}$ and $CD4^{-}CD8^{+}CD3^{lo}ROR\gamma t^{+}$ and $CD4^{-}CD8^{+}CD3^{+}ROR\gamma t^{+}$ T cells are shown. Data are shown as mean + SEM (n=18) from three independent experiments. Two-Way ANOVA plus Bonferroni post-tests to compare each column vs control column. Error bars, s.e.m.$^{*}P < 0.05$, $^{**}P < 0.01$, $^{***}P < 0.001$.
